# The modulatory role of gut microbiota on host behavior: exploring the interaction between the brain-gut axis and the neuroendocrine system

**DOI:** 10.3934/Neuroscience.2024004

**Published:** 2024-03-31

**Authors:** Temitope Awe, Ayoola Fasawe, Caleb Sawe, Adedayo Ogunware, Abdullahi Temitope Jamiu, Michael Allen

**Affiliations:** 1 Department of Cell Biology and Genetics, University of Lagos, Lagos, Nigeria; 2 School of Biological Sciences, Illinois State University, Normal, IL, USA; 3 Department of Neuroscience, Developmental and Regenerative Biology, University of Texas at San Antonio, San Antonio, TX, USA; 4 Department of Biomedical Sciences and Pathobiology, Virginia Tech, Blacksburg, VA, USA; 5 Department of Physiology, College of Medicine, Lagos State University, Lagos, Nigeria

**Keywords:** microbiota, brain-gut axis, behavior, microbiome, neuroendocrine, metabolites

## Abstract

The brain-gut axis refers to the communication between the central nervous system and the gastrointestinal tract, with the gut microbiome playing a crucial role. While our understanding of the interaction between the gut microbiome and the host's physiology is still in its nascent stage, evidence suggests that the gut microbiota can indeed modulate host behavior. Understanding the specific mechanisms by which the gut microbiota community modulates the host's behavior remains the focus of present and future neuro-gastroenterology studies. This paper reviews several pieces of evidence from the literature on the impact of gut microbiota on host behavior across animal taxa. We explore the different pathways through which this modulation occurs, with the aim of deepening our understanding of the fascinating relationship between the gut microbiome and the central nervous system.

## 1. Introduction

The brain-gut axis plays crucial roles in monitoring and harmonizing gut functions, being significantly influenced by the diverse symbiotic microbes housed within the gut of the host [1],[2]. The gut microbiota, consisting of trillions of microorganisms, has emerged as an important player in modulating host physiology. Its influence has been implicated in the pathogenesis of several disorders, including celiac disease and various neurological conditions [3]–[5]. With a growing body of studies in this exciting research area, it has become evident that these microbial communities exert a significant influence on various aspects of host behavior [6]–[12]. However, the mechanisms by which the gut microbiota mediates neuronal changes and behavioral modulations remain to be fully resolved.

In this review, our objective is to offer an overview of the influence of the gut microbiota on behavior, while highlighting the mechanisms through which the gut microbiota regulates behavior. Specifically, we emphasize the role of bacterial metabolites as neuromodulators. Additionally, we provide evidence for how the gut microbiota impacts the development of brain structures that drive behavior. Moreover, we examine the influence of gut microbes on the development of specific brain structures that are involved in certain behaviors. Ultimately, we conclude that further studies are still needed to unravel in detail how the brain-gut-microbiota (BGM) axis interacts to modulate host behavior.

## 2. The gut microbiota

Over the years, microbiota communities have been identified in animals across different taxa, including nematodes, insects, and most vertebrates [13],[14]. In humans, the gut microbiota, with tens of trillions of microbes, is by far the largest collection of microorganisms in the body [15]–[18]. Most of these microbes are bacteria that live in the colon and form symbiotic relationships with the host. Fungi, archaea, and viruses have also been reported to be present in the gut, but their underlying functions are not well known [19],[20]. Several experimental and clinical studies have shown that the gut microbiota has a significant impact on the physiology of the host, and it has been suggested to act via several pathways. Perhaps the most studied of these pathways is the brain-gut axis.

The brain-gut axis network includes the central nervous system (CNS), autonomic nervous system (ANS), enteric nervous system (ENS), and hypothalamic-pituitary-adrenal (HPA) axis [21]. The ANS, consisting of the sympathetic and parasympathetic limbs, conveys afferent and efferent signals between the intestinal lumen through the enteric, spinal, and vagal pathways to the CNS. The afferent signaling of the brain-gut axis is influenced by the microbial community in the gut [1]. Several studies have demonstrated that disruption of the gut microbiota can lead to several pathophysiological symptoms, many of which also lead to altered behavioral patterns [10],[12],[22]. It remains unresolved why and how these microbes influence the behavior of their hosts.

## 3. Microbiota and behavior

Evidence from animals across various taxa has demonstrated the modulatory influence of the gut microbiota on host behavior (Table 1). Behavioral changes attributed to the gut microbiota have been reported in various forms. In some cases, these changes manifest as alterations in feeding preference or orientation. For instance, in the nematode worm *Caenorhabditis elegans*, behaviors such as taxis (movement in response to specific stimuli) along chemical, temperature, or light gradients have been extensively studied. It has been observed that the ingestion of nutritive bacteria leads to a learned attraction and exploitation of the bacterial food source, while infection leads to the development of aversive behavior [23].

In *Drosophila*, the appetite for yeast as a food source is influenced by factors such as mating and dietary deprivation of essential amino acids. Interestingly, the presence of commensal bacteria in the fly's gut can completely suppress the appetite for yeast, even without affecting the levels of essential amino acids [7],[11]. This suggests that the modulation of host behavior by the gut microbiota is not solely attributed to bacteria providing essential nutrients but likely involves a complex interplay between the gut microbiota and the host neuroendocrine system.

Recent studies in mammals have provided evidence for the role of the gut microbiota in modulating host feeding decisions. For instance, germ-free mice colonized with different microbiomes from wild rodents, each exhibiting distinct natural feeding strategies, have exhibited significant variations in their voluntary dietary choices [24]. Furthermore, studies involving antibiotic-treated mice have demonstrated that the restoration of gut microbiota can reverse the overconsumption of high-sucrose pellets, indicating the direct impact of the microbiome on feeding behavior [25].

Mouse models of anorexia nervosa, a disorder characterized by self-starvation and weight loss, have shown disruptions in gut microbiome composition [26]. These imbalances suggest a potential link between gut dysbiosis and the pathogenesis of anorexia nervosa. Moreover, clinical studies have also reported gut dysbiosis in patients with anorexia nervosa, further supporting the involvement of the gut microbiota in the disorder's development [27],[28]. These findings highlight the significance of the gut microbiota in influencing feeding behaviors and their potential implications for understanding and treating eating disorders.

**Figure 1.**
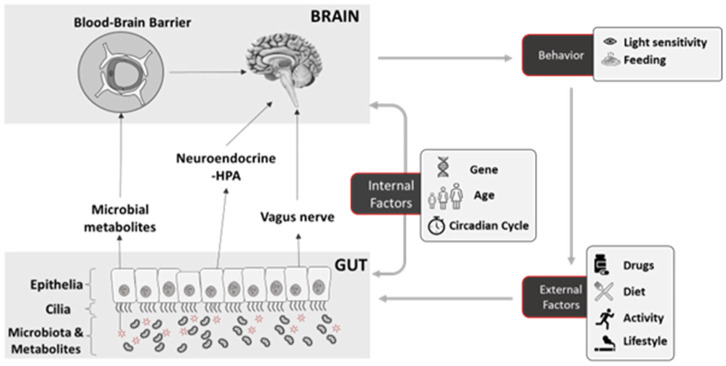
Simple schematic showing the different pathways used by the gut microbiota in altering the brain activities that drive behavioral changes, as well as the internal (physiological) and external (environmental) factors that can influence the host's microbiome balance.

The influence of the gut microbiota on the host extends to stress-related behaviors, with the involvement of the HPA axis as a key pathway in the BGM axis (Figure 1). Dysregulation of the BGM axis can contribute to stress, anxiety, sadness, and other mental conditions. For instance, prolonged exposure to stress inhibits egg laying in *C. elegans* [29],[30], and dysregulation of the gut microbiome has been found to impact egg-laying behavior in *C. elegans*. In a study by Gohari et al. [30], worms fed with different strains of non-pathogenic commensal bacteria, such as *Comamonas* or *Bacillus*, exhibited significant variations in egg retention, suggesting stress-related behavioral changes due to the gut microbiota.

In birds, feather pecking (FP) is a stress-induced neuropsychological disorder thought to result from disrupted communication between the gut and the brain. A study by Mindus et al. revealed that administering probiotics promoting *Lactobacillus rhamnosus* colonization of the gut helped mitigate stress-induced FP in White Leghorn laying hens [31]. This finding highlights the potential of modulating the gut microbiota to influence stress-related behaviors in avian species.

In mammals, *Bacteroides* species have shown promising results in improving repetitive and anxiety-like behaviors as well as communicative impairments, potentially through the restoration of specific bacterial metabolites [9],[32]. *Lactobacillus* treatments have been associated with improvement of prosocial behavior in mice, potentially through the regulation of oxytocin [9],[33].

The role of the BGM axis in the etiology of schizophrenia has also been investigated in humans. Zhu et al. reported that alterations in tryptophan-kynurenine metabolism, influenced by changes in the gut microbiota composition, may play a role in the development of schizophrenia [34]. Furthermore, the administration of psychobiotics (probiotics with potential mental health benefits) in humans has shown positive impacts on anxiety-like behavior [35],[36].

**Table 1.** Impact of gut microbiota on behavior across diverse animal species.

**Animal species**	**Affected behavior**	**Source**
Nematodes (*C. elegans*)	Stress related (egg laying behavior); feeding preference	[23],[37],[38]
Fruit flies (*D. melanogaster*)	Feeding preference	[7],[11]
Mice (*M. musculus*)	Social behavior; feeding behavior	[24],[25],[26],[27],[28],[32],[33]
Birds (*G. domesticus*)	Stress related (feather pecking)	[31]
Humans (*H. sapiens*)	Stress related (schizophrenia)	[35],[36]

Several hypotheses have been proposed to elucidate why the gut commensal community may exert influence over the behavior of their hosts, the foremost of which is the behavioral manipulation hypothesis. The behavioral manipulation hypothesis suggests that microbes have evolved strategies to modify the behavior of their hosts, ultimately benefiting their own survival and transmission [39]. This phenomenon is observed in various contexts, including the interaction between commensal *Providencia* bacteria and *C. elegans*, where the bacteria influence the host's food selection preference [38]. Similarly, in parasitic infections such as rabies, infection-induced inflammation of the CNS leads to increased aggression in the host (dogs or human), facilitating the transmission of the parasite [9]. These examples highlight the ability of gut microbes to manipulate host behavior, potentially driven by evolutionary adaptations to promote their own fitness.

Despite a growing body of work corroborating the modulatory influence of the gut microbiota on behavior, the precise mechanisms by which the gut microbiota mediate neuronal changes that drive behavioral modulations, including alterations in brain structure, connections, neurochemistry, and the endocrine system, remain to be fully elucidated.

## 4. How does microbiota regulate the host's behavior?

The gut is intricately connected to the CNS through various pathways, which likely play prominent roles in how the gut microbiome influences the brain and directs the behavior of the host (Figure 1). There is a growing body of evidence supporting these connections. Particularly noteworthy are studies investigating how bacterial metabolites can act as neuromodulators in the host's CNS [40],[41]. These signaling molecules can act on nearby vagus nerves that travel from the gut to the CNS [42]–[44]. The gut and the CNS are connected by the vagus nerve. The nerve can detect mechanical and chemical stimuli within the gut and subsequently transmit the signal to the brain for integration and responses. These responses could manifest as behavioral changes such as appetite control or emotion regulation, or they may take the form of direct feedback to the gut such as altering the gut microbial communities [42]–[44]. Indeed, blocking the vagal pathway through subdiaphragmatic vagotomy (SDV) altered gut microbiota–induced depression-like states in mice colonized with stress-inducing microbiome [43],[44]. This suggests that the vagus nerve plays a crucial role in driving the effect of gut microbiota on the brain (Figure 1).

Bacterial metabolites can also be released into the bloodstream, directly influencing distant targets in the brain or potentially compromising the blood–brain barrier, allowing non-local modulators to act on brain targets [45],[46].

Additionally, the composition of the gut microbiota during post-embryonic development can impact brain development [47]–[50]. By altering the development of certain brain structures, particularly those upstream of the hypothalamic-pituitary-adrenal axis (Figure 1), the gut microbiota can have a lasting consequence on certain behaviors later in life.

## 5. Bacteria metabolites and host behavior

Short-chain fatty acids (SCFAs), such as acetate, propionate, and butyrate, have been implicated in the regulation of neuro-immuno-endocrine processes, and their potential influence on host behavior have been highlighted [51]. SCFAs are primarily produced through bacterial fermentation of dietary fibers in the colon, and their effects on appetite and energy balance have been of particular interest.

One aspect of SCFA-mediated modulation of behavior is their interaction with the ghrelin signaling pathway. Ghrelin, an orexigenic hormone produced in the gastrointestinal tract, plays a role in regulating energy balance and promoting food intake. Studies have shown that SCFAs can bind to GHSR-1, a receptor for ghrelin, and modulate the effect of ghrelin on food intake. The presence of SCFAs has been found to reduce calcium influx in response to ghrelin stimulation in vitro, suggesting a potential inhibitory effect on ghrelin signaling [52].

Furthermore, SCFAs, particularly acetate, have been shown to influence appetite and food intake through their effects on the brain. Acetate has been reported to reduce appetite and increase satiety by acting as a signaling molecule in the CNS [53]. These findings suggest that SCFAs can potentially impact the host's feeding behavior by modulating neuroendocrine signaling pathways.

In *C. elegans*, the commensal bacteria *Providencia* has been found to produce the neuromodulator tyramine. This neuromodulator can bypass the need for host tyramine biosynthesis to ultimately modulate the aversive olfactory response mediated by ASH neurons via octopamine signaling. Interestingly, worms colonized by *Providencia* demonstrate a preference for these bacteria in food choice assays, and this preference depends on the presence of tyramine produced by the bacteria [38]. These findings suggest the ability of gut commensals to generate neuromodulators that directly influence the behavior and food preferences of their host organism, thereby facilitating the colonization and proliferation of their own species.

Studies in germ-free mice have provided valuable insights into the influence of the gut microbiota on hormones and neurotransmitters, highlighting the role of microbial modulation in neurochemistry. An example is the effect of gut microbial colonization on serotonin levels. Germ-free mice have been shown to exhibit decreased circulating serotonin compared to wildtype mice. However, when these germ-free mice were colonized with specific spore-forming bacteria from the mouse and human microbiota, serotonin levels were elevated, suggesting that these bacteria promote the biosynthesis of serotonin in the colonic enterochromaffin cells [54]. This suggests the capability of certain microbial strains to influence serotonin production.

Furthermore, germ-free mice have been observed to have reduced serotonin receptors in brain regions such as the amygdala and hippocampus, which are involved in emotional and cognitive processes [42],[55]. Changes in neurotransmitter turnover have also been observed, with an increased turnover of serotonin and dopamine in the striatum of germ-free mice [56]. These findings suggest that the presence or absence of a diverse gut microbiota can have profound effects on the neurochemistry of the host.

Bacterial-derived enzymes, such as caseinolytic protease B (ClpB), have been found to mimic the action of alpha-melanocyte-stimulating hormone (α-MSH), which can enhance the expression of satiety hormones like peptide YY (PYY) and Glucagon-like peptide 1 (GLP-1) or directly activate anorexigenic neurons. This signaling then promotes feelings of satiety, potentially contributing to the regulation of appetite [6],[10],[12]. This finding suggests a potential mechanism through which gut microbiota may influence the loss of appetite. It would be interesting to investigate whether the involvement of the gut microbiota in conditions like anorexia is mediated through the α-MSH signaling pathway.

Taken together, these findings show that bacterial-derived metabolites can influence the production of neuroactive compounds including several neurotransmitters, neurohormones, anti-inflammatory cytokines, and stomach endocrines, which are capable of driving the modulation of neuronal functions. This can ultimately inform alterations in the behavior of the hosts.

## 6. Microbiota and brain development

Gut microbiota can impact brain development and alter the organization of specific brain regions responsible for social behavior, stress response, and homeostasis. The amygdala, which plays a significant role in social behavior and anxiety, can be affected by the gut microbiota. Germ-free mice demonstrated enlarged amygdala and dendritic hypertrophy, particularly in the basolateral amygdala (BLA) [48]. Dendritic hypertrophy in the BLA is associated with changes in stress response and can contribute to conditions such as posttraumatic stress disorder (PTSD). Studies have also demonstrated that germ-free mice show an expansion of their hippocampus compared to control mice. Furthermore, the morphology of hippocampal pyramidal neurons, which are essential for learning and memory processes, has been found to be altered in germ-free mice [50]. These neurons in germ-free mice exhibit shorter and less branched structures compared with those in mice with a conventional microbiota [47],[50]. The hippocampus plays a crucial role in regulating stress response and emotional processing, being involved in the coordination of the HPA axis, which is responsible for the release of stress hormones. The alterations observed in the amygdala and hippocampus of germ-free mice suggest that the gut microbiota may contribute to the development and maturation of these brain regions, potentially influencing the individual's behavioral responses to stress later in life through the HPA axis (Figure 1).

Brain regions involved in cognitive and executive decision-making processes have also been shown to be influenced by the gut microbiota. Studies have revealed that germ-free mice and mice treated with antibiotics exhibit hypermyelination and increased expression of genes involved in myelination and myelin plasticity in the prefrontal cortex, a region associated with cognitive and executive decision-making [47],[57]. Similar observations have been made in adult animals treated with antibiotics, indicating that the gut microbiota may play a role in myelination processes in the brain [58].

Additionally, perturbation of the gut microbiota during adolescence using antibiotics has been shown to result in reduced brain cytokines and interleukin (IL)-6 levels in adulthood, along with impairment in object recognition in mice [49]. These findings highlight the critical role of the gut microbiota during early brain development and its potential impact on the normal development of cognitive functions.

## 7. Factors that influence the gut microbiota

The composition of the gut microbiota is dynamic and varies among individuals and even within the same individual at different times. This variation is influenced by various factors including genetics, mode of delivery during birth, age, hygiene, health, hormonal status, diet, lifestyle habits, and circadian cycle [46],[59]–[61] (Figure 1).

An interplay between genetic variation and gut microbiota has been supported by multiple studies as specific genetic loci have been linked to gut microbiota variation. For instance, in a study of ~6,000 European individuals, variations in the lactase-producing gene LCT were strongly correlated with *Bifidobacteria* populations. As such, lactose-intolerant individuals tend to have a larger population of *Bifidobacterium* in the gut microbiota [62].

Dietary habits, such as nutrient composition, mealtimes, and food behaviors, have a more chronic effect on the gut microbiota than short-term dietary interventions [60]. Extensive antibiotic use and gut microbiota profiles are closely related. For instance, in children suffering from late-onset autism, there is a correlation between antimicrobial therapy, gastrointestinal abnormalities, and significant presence of certain bacterial species [63]. Elderly hospitalized individuals often experience chronic alterations in their gut microbiota due to polypharmacy (taking multiple medications at once) [64].

Engaging in physical exercise has also been associated with increased intestinal biodiversity [65],[66], while heavy alcohol consumption significantly reduces the biodiversity of the gut microbiota [46],[67]. Additionally, the composition and function of the gut microbiota varies with the daily rhythm [61],[68], suggesting the circadian cycle to be an important modulating factor for the composition of the gut microbiome.

The structure of the gut lining also affects how symbiotic microbes are cleared and recruited. For instance, the internal mucociliary epithelium facilitates the recruitment of symbiotic bacteria by creating two well-defined flow fields: one that actively filters bacteria-sized particles from the ambient flow into a sheltered zone and another that provides the sheltered zone with enhanced diffusion, such that biochemical signaling between bacterial candidates and host epithelial cells may be facilitated [69].

Lastly, microbiota is not just found in the gut. Accessory gastrointestinal organs like the mouth, bladder, and distant organs like the lungs and vagina have all been reported to have microbial colonization. Evidence of crosstalk between the various microbiota communities is beginning to emerge [70], indicating that an imbalance in the microbiota of one organ could impact the balance of the gut microbiota and, in turn, the behavior of the host.

These findings demonstrate that the gut microbiota is influenced by a broad range of factors encompassing the host's genetics, physiological state, and environmental conditions. These, in turn, play a crucial role in modulating host behavior. Understanding these influences and their impact is crucial for advancing our knowledge of the mechanisms by which the gut microbiota modulates various aspects of host behavior.

## 8. Limitation

This minireview aims to gather evidence from the literature supporting the hypothesis that the gut microbiota influences host behavior. We acknowledge that certain studies may not have been included in our review due to various reasons, including publication language and accessibility, as well as studies published while this manuscript was under review. However, we provided evidence from the literature demonstrating the relationship between the gut microbiota and host behavior.

A significant general limitation among many articles included in this manuscript is the use of germ-free mice, administration of antibiotics, or colonization with wild microbes. While these techniques offer researchers a unique opportunity to investigate the roles of microbiota in behavior and various physiological processes, it is crucial to acknowledge certain challenges related to the methodology. Germ-free mice suffer severe developmental effects as well as inability to mount appropriate immune responses [71]. Similarly, the administration of antibiotics has been documented to impact the function of accessory gastrointestinal structures like the spleen [72],[73], potentially by disrupting the microbiota population within these organs. As a result, it is difficult to determine if a behavioral change is caused by gut microbiota signaling, bacteria from other organs, or simply the animals being disordered. Future research should focus on untangling this complexity.

## 9. Conclusion and future perspective

It is now obvious that the influence of the gut microbial community is not only restricted to the gut. It exerts modulatory effects on host behavior through complex interactions with the CNS via the brain-gut axis. Bacterial metabolites play key roles in mediating these effects. Genetic and environmental factors can affect gut microbiome composition and indirectly be responsible for the consequent behavioral changes. Additionally, disruption of the communication between the gut microbiota and the microbiota communities of other accessory and non-accessory organs may impact the behavior of the host. Future studies may clarify this crosstalk and its implication on the host's behavior.

Understanding the mechanisms underlying the microbiota-behavior relationship has important implications for various fields, including neurobiology, psychiatry, and the development of potential therapeutic interventions for behavioral and mental health disorders. Therefore, further research is needed to unravel the intricate connections between the gut microbiota and behavior, ultimately leading to a deeper understanding of the BGM axis.
